# Antigen-independent, autonomous B cell receptor signaling drives activated B cell DLBCL

**DOI:** 10.1084/jem.20230941

**Published:** 2024-03-21

**Authors:** Janneke A. Eken, Marvyn T. Koning, Kristyna Kupcova, Julieta H. Sepúlveda Yáñez, Ruben A.L. de Groen, Edwin Quinten, Jurriaan Janssen, Cornelis A.M. van Bergen, Joost S.P. Vermaat, Arjen Cleven, Marcelo A. Navarrete, Bauke Ylstra, Daphne de Jong, Ondrej Havranek, Hassan Jumaa, Hendrik Veelken

**Affiliations:** 1 Department of Hematology, Leiden University Medical Center, Leiden, Netherlands; 2 BIOCEV, First Faculty of Medicine, Charles University, Prague, Czech Republic; 3 First Department of Internal Medicine—Hematology, General University Hospital and First Faculty of Medicine, Charles University, Prague, Czech Republic; 4 School of Medicine, Universidad de Magallanes, Punta Arenas, Chile; 5 Department of Pathology, Amsterdam University Medical Center, Amsterdam, Netherlands; 6 Department of Pathology, Leiden University Medical Center, Leiden, Netherlands; 7 Institute of Immunology, University of Ulm, Ulm, Germany

## Abstract

Diffuse large B cell lymphoma of activated B cell type (ABC-DLBCL), a major cell-of-origin DLBCL subtype, is characterized by chronic active B cell receptor (BCR) signaling and NF-κB activation, which can be explained by activating mutations of the BCR signaling cascade in a minority of cases. We demonstrate that autonomous BCR signaling, akin to its essential pathogenetic role in chronic lymphocytic leukemia (CLL), can explain chronic active BCR signaling in ABC-DLBCL. 13 of 18 tested DLBCL-derived BCR, including 12 cases selected for expression of IgM, induced spontaneous calcium flux and increased phosphorylation of the BCR signaling cascade in murine triple knockout pre-B cells without antigenic stimulation or external BCR crosslinking. Autonomous BCR signaling was associated with IgM isotype, dependent on somatic BCR mutations and individual *HCDR3* sequences, and largely restricted to non-GCB DLBCL. Autonomous BCR signaling represents a novel immunological oncogenic driver mechanism in DLBCL originating from individual BCR sequences and adds a new dimension to currently proposed genetics- and transcriptomics-based DLBCL classifications.

## Teaser

This study describes and characterizes antigen-independent, autonomous signaling of the clonal B cell receptor as an intrinsic oncogenic driver in activated B cell type DLBCL. This long-sought non-genetic lymphomagenic mechanism has profound implications for development of effective novel therapies.

## Introduction

Diffuse large B cell lymphoma (DLBCL) is an aggressive malignancy of mature post–germinal center (GC) B cells (GCB) and the most frequently diagnosed hematologic malignancy (Alaggio et al., 2022; Campo et al., 2022). Standard immunochemotherapy with the rituximab plus cyclophosphamide, doxorubicin, vincristine, and prednisone (R-CHOP) regimen cures 23–74% of DLBCL patients, but around 35% of patients eventually succumb to their disease. Relatively poor survival of activated B cell type DLBCL (ABC-DLBCL) compared with GCB type DLBCL provides an urgent need for development of effective novel therapies that target critical driving pathways (Alizadeh et al., 2000; Howlader et al., 2017; Rosenwald et al., 2002).

Survival and growth of ABC-DLBCL cells are thought to be dependent on chronic active B cell receptor (BCR) signaling (Davis et al., 2010). In 10% of DLBCL, activating *CARD11* mutations mimic signaling originating from the BCR at a downstream level (Lenz et al., 2008; Wilson et al., 2015). In 21–25% of ABC-DLBCL, activating *CD79B *mutations provide proximal enhancement of BCR-mediated signaling but do not initiate BCR signaling themselves (Davis et al., 2010; Reddy et al., 2017; Schmitz et al., 2018; Wilson et al., 2015). In addition, up to 40% of ABC-DLBCL harbor a canonical *MYD88 *L265P mutation that cooperates downstream with BCR signaling in an NF-κB–activating supercomplex (Ngo et al., 2011; Phelan et al., 2018; Reddy et al., 2017; Schmitz et al., 2018). Because mutated CD79B and MYD88 are insufficient to activate the BCR signaling cascade themselves, the mechanism of activation of the BCR signaling cascade in ABC-DLBCL without activating *CARD11* mutations (*CARD11*^wt^ ABC-DLBCL) remains unclear, and an additional non-genetic driving mechanism has been postulated to induce the ABC-DLBCL phenotype (Wilson et al., 2015). BCR engagement of autoantigens has been shown in selected DLBCL cases, including binding of the BCR expressed by the ABC-DLBCL cell line TMD8 to itself (Thurner et al., 2021; Young et al., 2015). We here propose antigen-independent, autonomous BCR signaling as a major alternative mechanism for NF-κB activation in DLBCL. We have previously shown that this phenomenon is an indispensable driver in chronic lymphocytic leukemia (CLL) (Dühren-von Minden et al., 2012). Homotypic BCR interactions mediated by crucial amino acid residues form the structural basis for antigen-independent signaling in paradigmatic CLL subtypes as identified by crystallographic studies (Maity et al., 2020; Minici et al., 2017). In contrast to CLL with frequently unmutated BCR gene sequences, we postulated that ABC-DLBCL has acquired autonomous BCR signaling by somatic hypermutation in a GC reaction.

## Results and discussion

**Figure 1.**
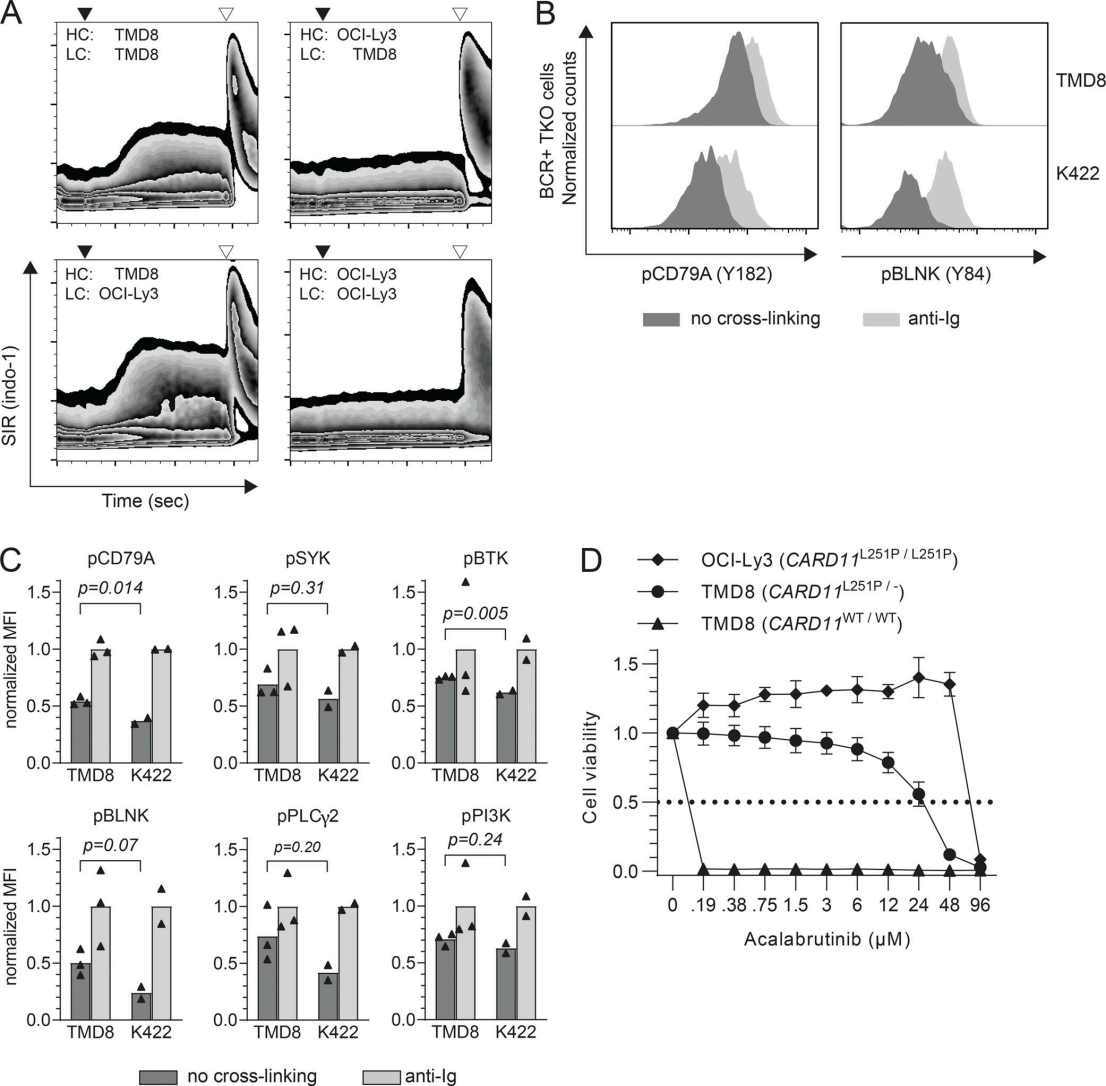
**Autonomous signaling activity of BCR of DLBCL cell lines. (A)** Calcium flux assay of TKO cell transduced with combinations of BCR heavy (HC) and light chains (LC) of ABC-DLBCL cell lines TMD8 (*CARD11*^*WT/WT*^) and OCI-Ly3 (*CARD11*^*L251P/L251P*^). Black triangles indicate administration of 4-OHT. Open triangles indicate anti-Ig heavy chain crosslinking. **(B)** Histograms of fluorescence intensity of APC-labeled CD79A phosphorylated on tyrosine (Y) 182 and BLNK phosphorylated at Y84 as measured by phospho-flow cytometry of 4-OHT–treated TKO cells transduced with the BCR of DLBCL cell lines TMD8 and Karpas 422 (K422). Light gray: anti-Ig heavy chain (anti-Ig) crosslinking. Dark gray: no BCR crosslinking. **(C)** Phospho-flow cytometry of 4-OHT–treated TKO cells transduced with the BCR of DLBCL cell lines TMD8 and Karpas 422. Triangles represent individual measurements. Bars represent MFI of AF647/APC after normalization to the MFI induced by BCR crosslinking with anti-Ig. MFI of cells without crosslinking were compared by unpaired *t* test. **(D)** Cell viability assay after 96 h of culture with increasing concentrations of the BTK-inhibitor acalabrutinib. Values were normalized to medium control. Error bars show standard deviation of duplicate measurements. Dotted line: IC50.

**Figure S1.**
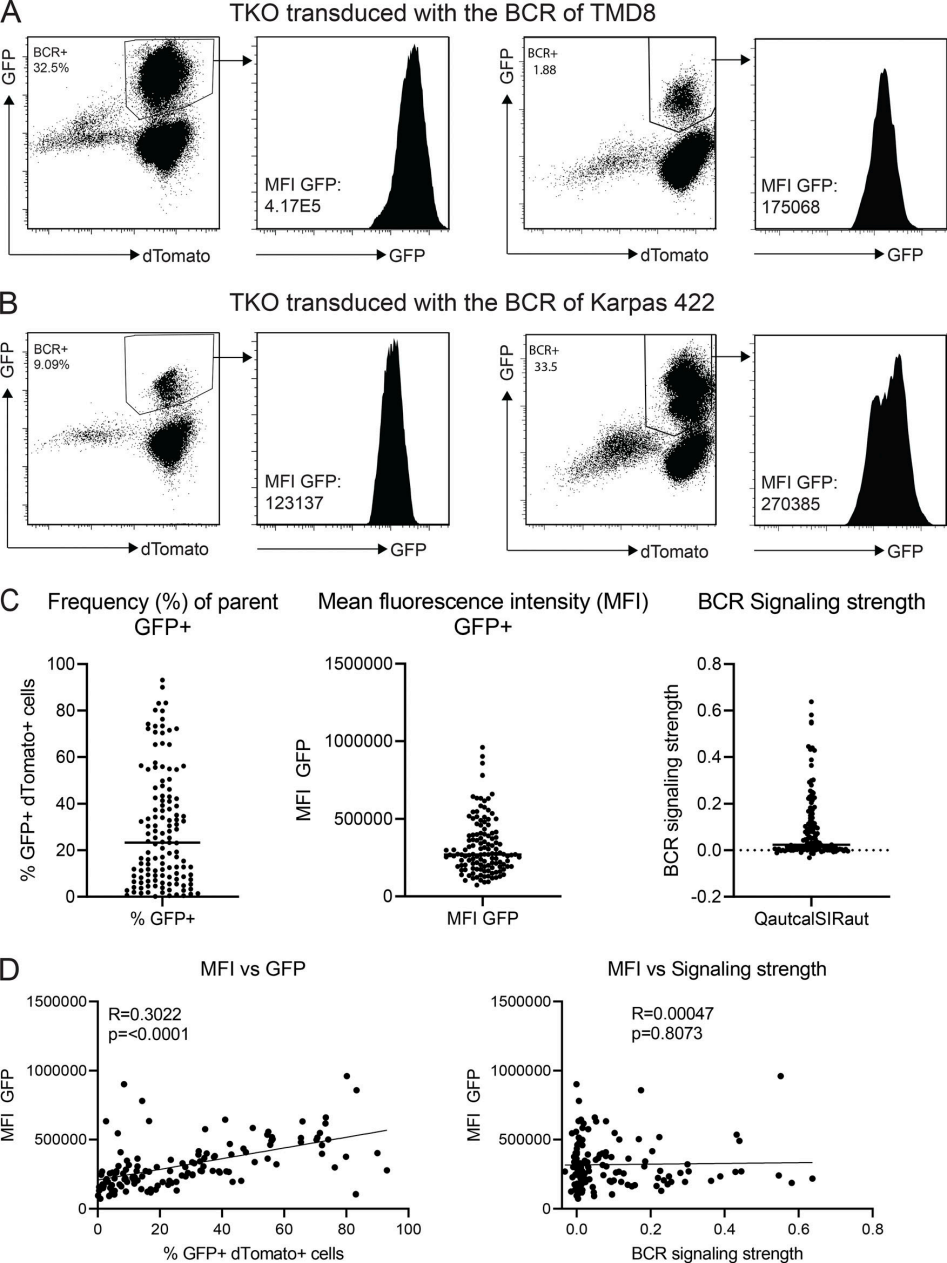
**Gating and quality control of flow cytometry. (A)** GFP expression on two TMD8 BCR transductants in TKO with high and low transduction efficiency showing gating for BCR-expressing cells. Histograms show MFI of GFP. **(B)** GFP expression on two Karpas 422 BCR transductants in TKO with high and low transduction efficiency showing gating for BCR-expressing cells. Histograms show MFI of GFP. **(C)** Distribution of GFP-positive cells, GFP MFI, and calculated BCR signaling strength of all samples among all experiments. Each dot represents one measurement. Horizontal lines indicate the median. **(D)** Correlations of GFP MFI with transduction efficiency and with BCR signaling strength. Transduction efficiency is correlated with GFP MFI. BCR signaling strength is not correlated to GFP MFI. Transduction efficiency and BCR expression do not influence the calculation of autonomous signaling strength of the BCR.

To explore this hypothesis in DLBCL, we transduced murine triple knockout (TKO) pre-B cells with functional BCR from the ABC-DLBCL cell lines TMD8 and OCI-Ly3 (Meixlsperger et al., 2007). TKO cells cannot express a pre-BCR and carry a tamoxifen-inducible version of the BCR signal transducer BLNK/SLP-65. Transduction of TKO cells with the TMD8 IgM κ BCR induced strong and sustained calcium flux without any BCR crosslinking (Fig. 1 A and S1). In contrast, TKO cells transduced with the OCI-Ly3 IgG λ BCR showed calcium flux only after external crosslinking with an anti-IgG antibody. Mutual exchange of the light chains between both BCR complexes indicated that antigen-independent BCR activation originates from the TMD8 Ig heavy chain (Fig. 1 A). Autonomous BCR signaling of the TMD8 BCR was also evident from enhanced phosphorylation of proteins of the BCR signaling cascade compared with the non-signaling BCR of the Karpas 422 cell line (Fig. 1, B and C). Consistent with dependency on active BCR signaling, TMD8 cells were highly sensitive to proximal BCR signaling blockade by the Bruton tyrosine kinase (BTK) inhibitor acalabrutinib (Fig. 1 D). In contrast, OCI-Ly3 cells carry a biallelic activating *CARD11* L251P mutation (Lenz et al., 2008) and were highly resistant to BTK inhibition. Introduction of the *CARD11* L251P mutation by CRISPR/Cas9-mediated homology-directed repair converted TMD8 cells to a highly acalabrutinib-resistant phenotype (Fig. 1 D). These results provided initial support for our hypothesis of autonomous BCR signaling and oncogenic *CARD11 *mutations as alternative but equivalent NF-κB–activating mechanisms in ABC-DLBCL.

**Figure 2.**
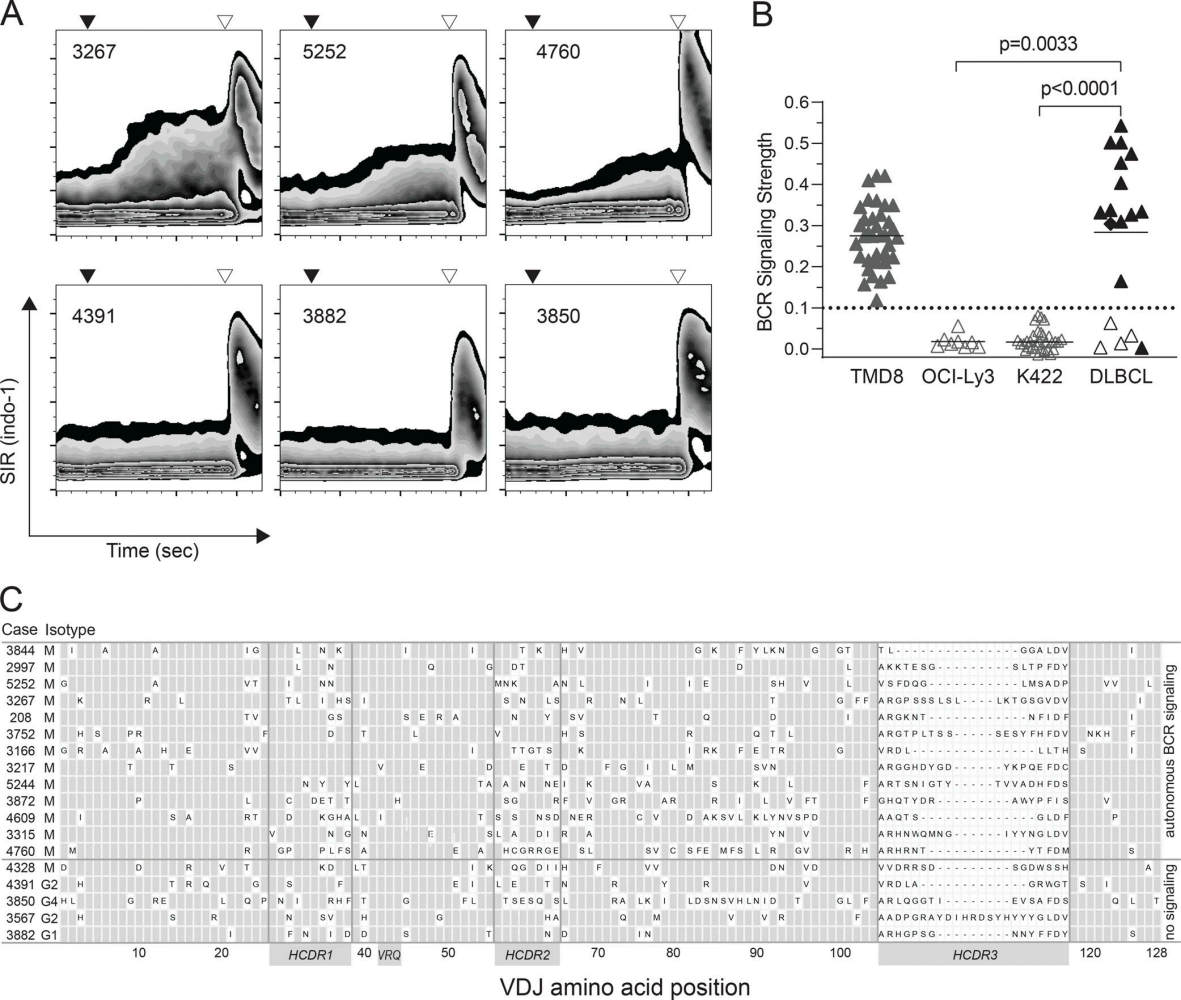
**Autonomous signaling of BCR from primary DLBCL cases. (A)** Calcium flux assay of TKO cells transduced with BCR from six primary DLBCL with (upper row) and without (bottom row) autonomous signaling. Black triangles indicate administration of 4-OHT. Open triangles indicate anti-Ig heavy chain crosslinking. **(B)** Signaling strength of BCR from primary DLBCL cases and DLBCL cell lines TMD8, OCI-Ly3, and Karpas 422 (K422). All measurements of the BCR from the DLBCL cell lines during this study are shown. Symbols represent arithmetic means of two consecutive measurements. Experimental groups were compared by Wilcoxon’s signed rank test. Open symbols: BCR of IgG isotype. Solid symbols: BCR of IgM isotype. Diamond: BCR from a DLBCL originating as transformation of a follicular lymphoma. The dotted line indicates the upper limit of BCR signaling strength of non-signaling BCR. **(C)** BCR characteristics of primary DLBCL cases. Letters indicate one letter code of amino acids acquired by SHM relative to predicted germ-line sequences in IMGT.

Next, we assessed autonomous BCR signaling in 18 cryopreserved biopsy samples of histopathologically confirmed DLBCL. Since our hypothesis was focused on ABC-DLBCL, known to express predominantly the IgM isotype (Ruminy et al., 2011), 12 of these biopsies were selected for expression of an IgM BCR based on informative immunohistochemistry. Sequencing of BCR transcripts showed a substantial mutational burden in all cases with a median 87% (range: 68–96%) *IGHV* homology to the germ-line allele (Table S1). 13 of 18 tested DLBCL BCR induced robust, antigen-independent calcium flux in transduced TKO cells (Fig. 2, A and B). All autonomously signaling DLBCL BCR were of IgM isotype (Fisher’s exact test: P = 0.0016). There was no recognizable association with somatic hypermutation burden and distribution between signaling and non-signaling DLBCL BCR (Fig. 2 C).

**Figure 3.**
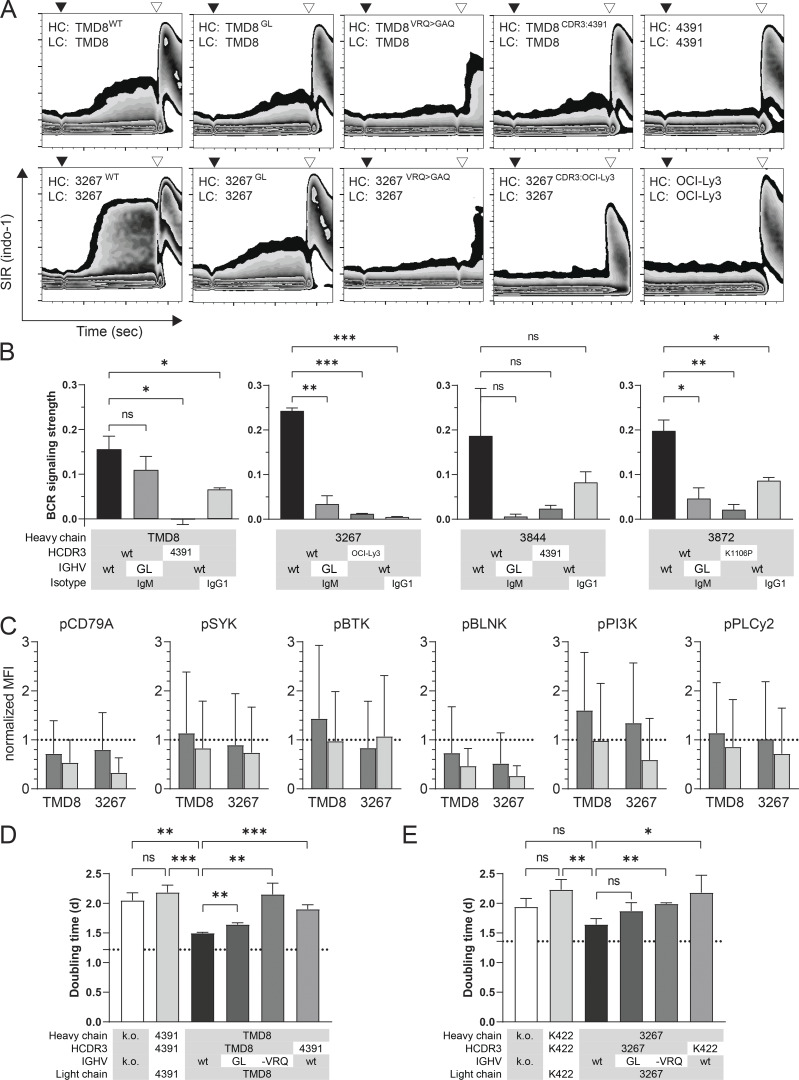
**BCR sequence characteristics required for autonomous signaling. (A)** Calcium flux assay of TKO cells transduced with wild-type or modified BCR of DLBCL cell lines TMD8 and DLBCL case 3267. HC: Heavy chain. LC: Light chain. wt: wild-type BCR expressed by cell lines and DLBCL biopsies. GL: *IGHV* of BCR reverted back to germ-line sequence. VRQ (valine-arginine-glutamine) > GAQ (glycine-alanine-glutamine): FR2 VRQ motif essential for autonomous BCR signaling reverted to GAQ. CDR3: 4391: TMD8 *HCDR3* replaced with *HCDR3* from DLBCL case 4391. CDR3:OCI-Ly3: 3267 *HCDR3* replaced with *HCDR3* from OCI-Ly3. Black triangles indicate administration of 4-OHT. Open triangles indicate anti-Ig heavy chain crosslinking. **(B)** Signaling strength of wild-type (wt) or modified BCR of DLBCL cell line TMD8 and DLBCL cases 3267, 3844, and 3872 as calculated from calcium flux assays of TKO cells. Modifications included reversion of *IGHV* back to its corresponding germ-line sequence (GL), replacing of the original *HCDR3* with *HCDR3* from closely related, non-signaling DLBCL BCR as indicated, and change of the constant region from IgM to IgG1. Bars represent mean of two repetitive measurements per sample. Comparisons between BCR signaling strengths were performed using one-sided unpaired *t* test. **(C)** Phospho-flow cytometry of 4-OHT–treated TKO cells transduced with wild-type or modified BCR of DLBCL cell line TMD8 or DLBCL case 3267. Bars represent MFI of AF647/APC after normalization to the MFI induced by BCR crosslinking with anti-Ig. The dotted line indicates the relative MFI measured after anti-Ig crosslinking. Dark bars: wild-type BCR. Bright bars: BCR reconverted to germ-line sequence. Error bars indicate standard deviation of AF647/APC MFI of all measured cells. Comparisons by unpaired *t* test showed P < 0.0001 between wild-type and GL BCR for all proteins. **(D)** Growth support assay of CRISPR-modified U2932 cells with BCR from TMD8 and DLBCL 4391. Proliferation is measured as cellular doubling time. k.o.: BCR KO. wt: wild-type TMD8 *IGHV*. GL: TMD8 *IGHV* reverted back to germ-line sequence. -VRQ: FR2 VRQ motif essential for autonomous BCR signaling reverted to GAQ. Dotted line indicates mean doubling time of unmodified wild-type U2932 cells. Error bars represent variation of triplicate measurements. Comparisons between experimental groups were performed using two-sided unpaired *t* test. **(E)** Growth support assay of CRISPR-modified U2932 cells with BCR from Karpas 422 (K422) and DLBCL 3267. Proliferation is measured as cellular doubling time. k.o.: BCR KO. wt: wild-type 3267 *IGHV*. GL: 3267 *IGHV* reverted back to germ-line sequence. -VRQ: FR2 VRQ motif essential for autonomous BCR signaling reverted to GAQ. Dotted line indicates mean doubling time of unmodified wild-type U2932 cells. Error bars represent variations of triplicate measurements. Comparisons between experimental groups were performed using two-sided unpaired *t* test. *, P < 0.05; **, P < 0.01; ***, P < 0.001; ns, not significant.

**Figure S2.**
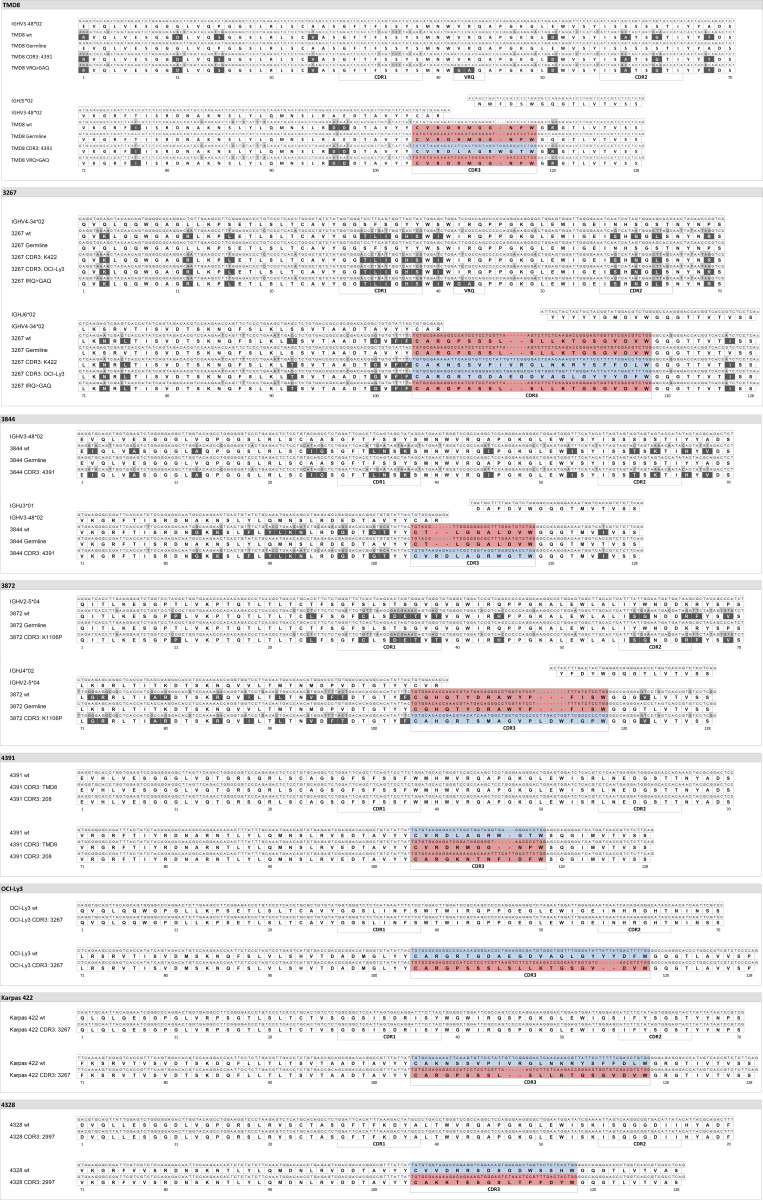
**Sequence alignment of closely related and modified V and J sequences used for functional testing.** Light gray boxes indicate nucleotide variants to the germ-line sequence. Dark gray boxes indicate amino acid changes to the germ-line sequence. Red boxes indicate the *HCDR3 *sequence of the wild-type BCR. Blue boxes indicate an inserted alternative but closely related *HCDR3 *sequence. wt, wild-type.

We then investigated the dependency of autonomous BCR signaling on specific BCR sequence elements. To assess the contribution of somatic hypermutation (SHM) to autonomous signaling activity, *IGHV* genes of TMD8 and three DLBCL cases were reverted to their predicted germ-line sequences. All revertants retained full BCR functionality upon crosslinking, but autonomous BCR signaling was moderately reduced for TMD8 and strongly reduced for primary DLBCL BCR (Fig. 3, A and B). In the absence of external BCR crosslinking, consistently reduced phosphorylation of five of six investigated BCR signaling proteins was demonstrated by phospho-flow cytometry upon conversion of the TMD8 and case 3267 BCR to their *IGHV* germ-line sequence (Fig. 3 C). Next, we modified the canonical FR2 (V/I)RQ motif that is essential for homotypic BCR interactions in the majority of CLL (Dühren-von Minden et al., 2012) to a GAQ motif. This modification abolished autonomous signaling for TMD8 and DLBCL 3267 BCR but also impaired signaling after crosslinking. The predicted dependency of autonomous BCR signaling on individual heavy chain complementarity-determining region 3 (*HCDR3*) (Dühren-von Minden et al., 2012; Minici et al., 2017) was confirmed by replacing the *HCDR3* of TMD8 and of three primary DLBCL BCR with *HCDR3* from closely related BCR without autonomous signaling activity (Fig. 3 B and Fig. S2). To establish the observed association of autonomous signaling with the IgM isotype, we finally demonstrated consistent reduction of autonomous signaling strength when the BCR was expressed as IgG (Fig. 3 B).

The function of autonomously signaling BCR as a driver of lymphoma proliferation was independently confirmed in the ABC-DLBCL U2932 cell line (Fig. 3, D and E) (Havranek et al., 2017). Introduction of the autonomously signaling BCR of TMD8 and DLBCL 3267 by CRISPR/Cas gene editing enhanced U2932 proliferation as indicated by significantly reduced U2932 doubling times in comparison to non-actively signaling BCR. Removal of the internal (V/I)RQ motif and exchange of *HCDR3* with a non-signaling BCR reduced U2932 proliferation. *IGHV* sequence reversion to germline significantly prolonged cellular doubling time induced by the TMD8 BCR.

**Figure 4.**
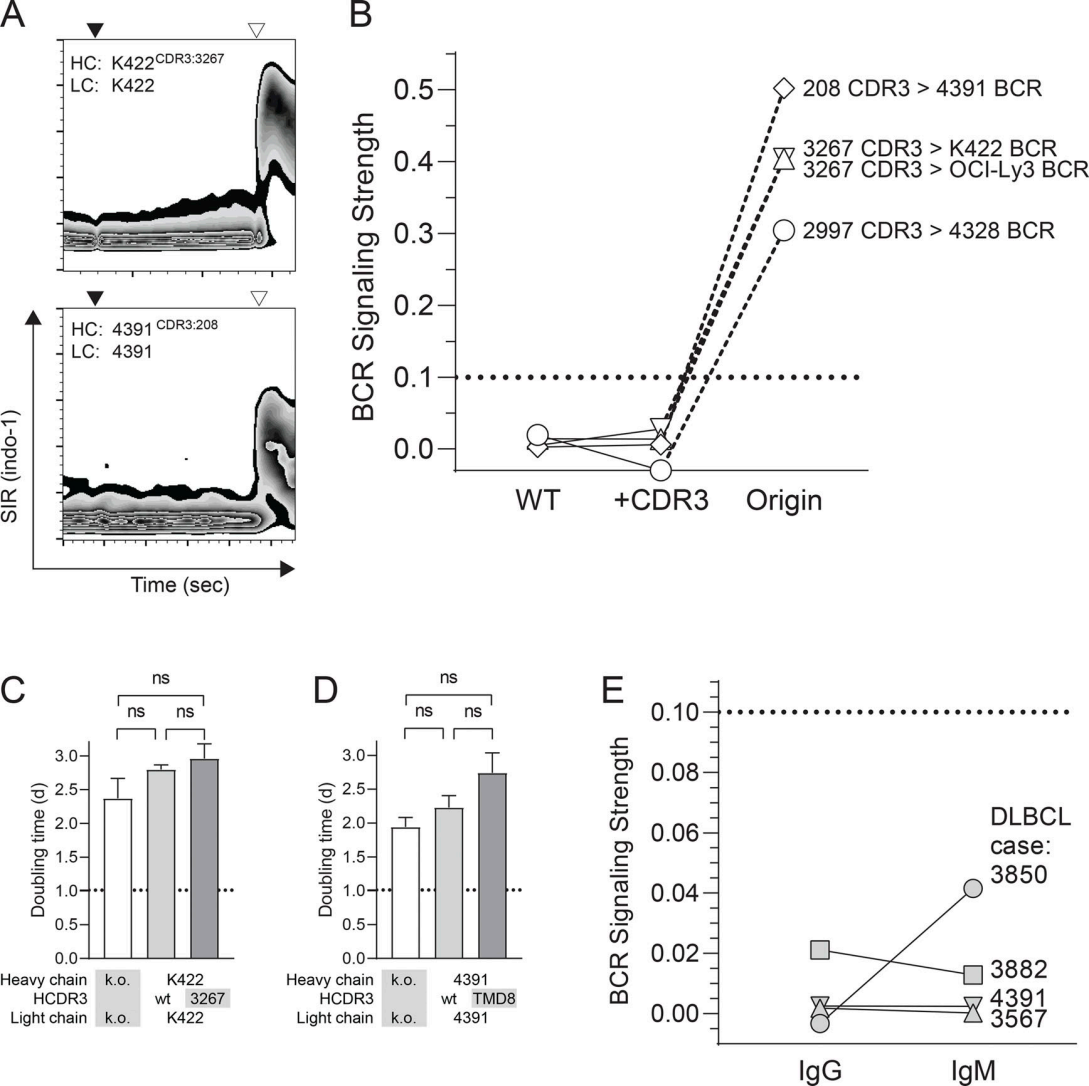
**Modifications to endow non-signaling BCR with autonomous signaling activity. (A)** Calcium flux assay of TKO cells transduced with modified BCR of DLBCL cell line Karpas 422 (K422) and DLBCL case 4391. BCR sequences were modified by *HCDR3* exchange with a closely related, autonomously signaling BCR as indicated. HC: Heavy chain. LC: Light chain. Black triangles indicate administration of 4-OHT. Open triangles indicate anti-Ig heavy chain crosslinking. **(B)** Signaling strength of non-signaling BCR after *HCDR3* exchange with an autonomously signaling BCR as calculated from calcium flux assays of TKO cells. Symbols represent arithmetic means of two consecutive measurements. Solid lines indicate identity of non-signaling BCR. Dashed lines indicate identity of *HCDR3*. WT: wild-type non-signaling BCR. +CDR3: Non-signaling BCR with exchanged *HCDR3*. The dotted line indicates the upper limit of BCR signaling strength of non-signaling BCR. **(C)** Growth support assay of CRISPR-modified U2932 cells with wild-type or modified BCR of DLBCL cell line Karpas 422 (K422). Proliferation is measured as cellular doubling time. k.o.: BCR KO. wt: wild-type K422 *HCDR3*. 3267: *HCDR3* replaced from closely related, autonomously signaling BCR from DLBCL case 3267. Comparisons between experimental groups were performed using two-sided unpaired *t* test. The dotted line indicates mean doubling time of unmodified wild-type U2932 cells. **(D)** Growth support assay of CRISPR-modified U2932 cells with wild-type or modified BCR of DLBCL 4391. Proliferation is measured as cellular doubling time. k.o.: BCR knock-out. wt: wild-type 4391 *HCDR3*. TMD8: *HCDR3* replaced from closely related, autonomously signaling BCR from cell line TMD8. Comparisons between experimental groups were performed using a two-sided unpaired *t* test. The dotted line indicates mean doubling time of unmodified wild-type U2932 cells. **(E)** Signaling strength of non-signaling BCR from primary DLBCL cases 3882 (IgG1), 4391 (IgG2), 3567 (IgG2), and 3850 (IgG4) to IgM as calculated from calcium flux assays of TKO cells. The dotted line indicates the upper limit of BCR signaling strength of non-signaling BCR.

Exchange of the *HCDR3* of non-signaling DLBCL BCR with the *HCDR3* of a closely related DLBCL BCR with autonomous signaling activity was insufficient to induce autonomous BCR signaling in TKO cells (Fig. 4, A and B) or to accelerate the growth of BCR-k.o. U2932 cells (Fig. 4, C and D). Likewise, expression of IgG DLBCL BCR as IgM isotype failed to facilitate autonomous signaling (Fig. 4 E).

**Figure 5.**
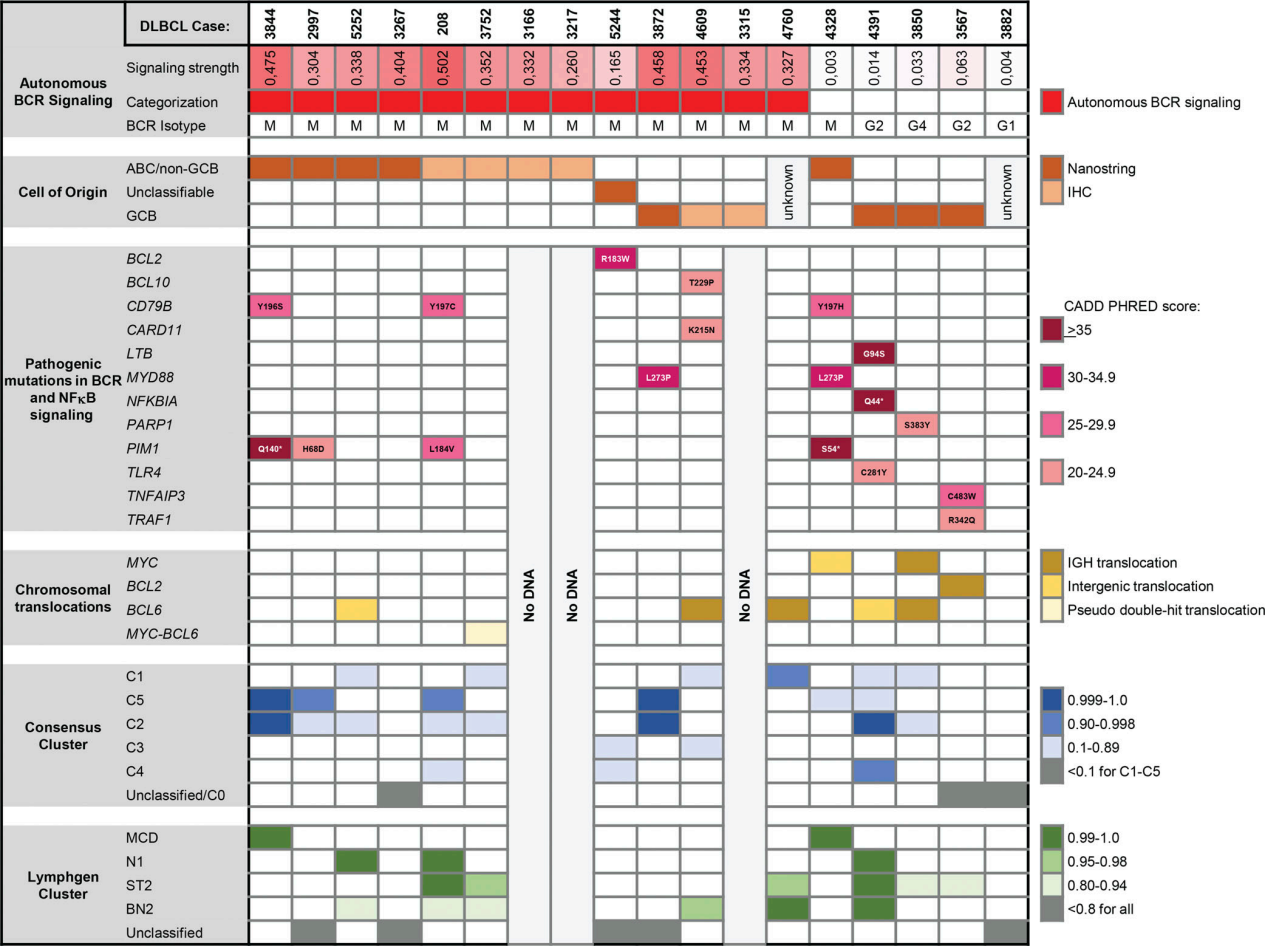
**Molecular characterization of primary DLBCL cases.** DLBCL cases are ordered according to categorical assignment to BCR with or without autonomous signaling and secondarily by COO classification. Cases are annotated for their expressed isotype, for *MYC*, *BCL2*, and *BCL6* translocations as detected by targeted sequencing, and for activating mutations in genes relevant for BCR signaling and NF-κB activation including *PIM1* detected by WES. The predicted protein sequence alterations are indicated. Only mutations annotated as “deleterious” by SIFT, “possibly” or “probably” damaging by PolyPhen, or “likely pathogenic” or “pathogenic” by Clinical Significance are shown. The probability of assignment to a molecular cluster is indicated per case. Assignment to consensus clusters was determined by probabilistic calculation. Assignment to LymphGen clusters was performed through the LymphGen data portal.

16 tested DLBCL were classified according to their cell of origin (COO) by digital gene expression profiling (*n* = 10) or immunohistochemistry (*n* = 6) (Hans et al., 2004; Scott et al., 2014). In accordance with our starting hypothesis, autonomous BCR signaling was predominantly observed in ABC-/non-GCB DLBCL (P <0.05; Fig. 5). Whole-exome sequencing (WES) and targeted sequencing of recurrent translocations were performed on the tested DLBCL cases to facilitate their assignment to proposed molecular clusters (Chapuy et al., 2018; Wright et al., 2020). Assignment to molecular clusters was generally hampered by ambiguous or weak assignment probabilities. Nevertheless, BCR of all five DLBCL assigned by probabilistic classification with a high likelihood (> 0.9) to the ABC-enriched consensus clusters C5 and C1 exhibited antigen-independent signaling (Fig. 5). Assignment of individual DLBCL to LymphGen clusters (Wright et al., 2020) was severely impaired by a large proportion of unclassifiable cases (Fig. 5).

Our data establish autonomous BCR signaling as a novel immunological driver mechanism in DLBCL. In CLL, autonomous BCR signaling frequently results from the *IGHV* structure generated at primary VDJ recombination without the necessity for SHM (Dühren-von Minden et al., 2012). In contrast, our data indicate that autonomous BCR signaling in DLBCL predominantly occurs via acquisition of nonsynonymous BCR mutations during GC passage. At present, detection of autonomous BCR signaling in DLBCL cannot be predicted by stereotypic BCR sequences as in CLL but requires individual functional testing for antigen-independent calcium mobilization or lymphoma growth acceleration in vitro. Similar to CLL, BCR stability and autonomous signaling in DLBCL are dependent on a heavy chain FR2 motif and the individual *HCDR3* sequence (Dühren-von Minden et al., 2012; Minici et al., 2017). The observed association of autonomous BCR signaling with the IgM isotype and hence the apparent necessity to avoid Ig class recombination in DLBCL deserves further mechanistic and structural examination.

Of note, cell-intrinsic autonomous BCR signaling differs fundamentally from BCR stimulation by external cognate self-antigen as proposed for DLBCL earlier (Thurner et al., 2021; Young et al., 2015). Cognate antigenic stimulation is dependent on the local concentration of the BCR-engaging antigen and its presence over time, even when the binding epitope is contained in a soluble form of the BCR. In contrast, DLBCL cells generally remain dependent on the continuous expression of a functional BCR complex (Davis et al., 2010). When this BCR complex has autonomous signaling activity, the cells are “condemned” to continuously receive this cell-intrinsic oncogenic signal. Autonomous BCR signaling might therefore offer a plausible, non-genetic explanation for the well-established dependence of ABC-DLBCL on an activated BCR pathway and constitutive NF-κB activation (Davis et al., 2001, 2010). Since recurrent *CD79* and *MYD88* mutations are assumed not to fully mimic BCR signaling themselves, it can be envisioned that they might act as important enhancers of the autonomous BCR signal toward malignant transformation (Davis et al., 2010; Ngo et al., 2011; Schmitz et al., 2018). This proposition is indirectly supported by clinical observations during pharmacological BTK inhibition, where high response rates to ibrutinib were seen in ABC-DLBCL with (55.5%) and without (31%) *CD79B* mutations, while oncogenic *CARD11* mutations downstream of BTK and NF-κB activation via genetic *TNFAIP3* inactivation were associated with resistance to ibrutinib (Wilson et al., 2015). Most recently, a post-hoc analysis of the Phoenix trial showed a beneficial effect of adding ibrutinib to R-CHOP for LymphGen *MYD88*^*L265P*^*/CD79B* (MCD) and *NOTCH1 *(N1) clusters (Wilson et al., 2021). These clusters are highly enriched for ABC-DLBCL, and MCD in particular for *CD79B* mutations and NF-κB activation (Schmitz et al., 2018). Within these clusters, activating mutations in downstream BCR signaling components are relatively rare and suggest an upstream origin of NF-κB activation, with autonomous BCR signaling as a potential candidate mechanism. Although three DLBCL with autonomous BCR signaling were indeed MCD/N1 cases in our experiments, it is impossible to establish a definite association between genetic clusters and BCR function since only 50–70% of DLBCL cases can reliably be assigned to LymphGen clusters at present (Mendeville et al., 2022; Schmitz et al., 2018). With respect to consensus clusters, all five cases assigned to the ABC-dominated clusters C5 and C1 showed autonomous BCR signaling. The only ABC-DLBCL (case 4328) that lacked autonomous BCR signaling was assigned to the MCD cluster and carried concurrent mutations in *CD79B *and *MYD88*. No additional NF-κB–activating mutations including in *CARD11* were found. Therefore, this particular case is in apparent contradiction to our simplistic model of autonomous BCR signaling or strong NF-κB–activating mutations as indispensable alternatives. However, this case also carried a sporadic frameshift mutation in the serine/threonine kinase *PIM1*. *PIM1* mutations are most prevalent in the usually ibrutinib-sensitive DLBCL MCD cluster but confer resistance to BTK inhibition in experimental systems and clinical trials (Kuo et al., 2016; Schmitz et al., 2018; Wilson et al., 2021). PIM1 inhibition has been suggested as a distinct therapeutic strategy in appropriately selected cases (Brault et al., 2012).

In conclusion, we have identified autonomous BCR signaling as a novel, potentially critical immunological driver in DLBCL. Autonomous BCR signaling appears to act as an oncogenic mechanism unless BCR signaling is mimicked by acquisition of activating mutations in downstream BCR signaling or of alternative genetic mechanisms to activate NF-κB. Therefore, intrinsic active BCR signaling adds an additional pathogenetic layer to genetics and transcriptomics in DLBCL. This oncogenic mechanism cannot be reliably detected by sequencing studies or gene expression profiling at present, requiring functional BCR testing to identify directly BCR-driven individual cases. As an important clinical implication, DLBCL cases that depend on autonomous BCR signaling are predicted to respond to pharmacological BCR signaling inhibition. The discovery of this previously postulated (Wilson et al., 2015) non-genetic oncogenic driver broadens our understanding of the malignant transformation of mature B cells and potentially refines the identification of candidate patients for BCR signaling inhibition in DLBCL.

## Materials and methods

### Cell lines and biopsies

Fresh-frozen biopsies of histologically confirmed DLBCL samples were identified in the pathology archive at Leiden University Medical Center (LUMC). The study was approved by the Scientific Review Committee of the LUMC Department of Hematology under an applicable waiver of consent by the LUMC Ethical Committee (B16.048).

DLBCL cell lines OCI-Ly3 (RRID:CVCL_8800), TMD8 (RRID:CVCL_A442), and U2932 (RRID:CVCL_1896) were obtained from the German Collection of Microorganisms and Cell Cultures. Phoenix cells (RRID:CVCL_H717) were cultured at 5% CO_2_ in Iscove’s Modified Dulbecco’s Medium (IMDM; Lonza) supplemented with 10% fetal bovine serum (FBS; Bodinco), 3 mM *L*-glutamine (Lonza), 100 U/ml penicillin/streptomycin (Lonza). TKO cells were used, which are generated from the *Oct* cell line (RRID:CVCL_WN86) that originates from transformed murine pre-B cells from *Slp65*^−/−^ mice. The TKO cell line has a *Rag2* and *Lambda5* KO. These cells were cultured at 7% CO_2_ in Iscove’s Basal Medium (Merck) supplied with 5% FBS (PAN BioTech), 3 mM *L*-glutamine, 100 U/ml penicillin/streptomycin, 50 mM 2-mercaptoethanol (Sigma-Aldrich; Merck), and IL-7–containing supernatant of mouse plasmacytoma cells J558L (RRID:CVCL_3949), stably transfected with a murine IL-7 expression vector (Meixlsperger et al., 2007).

### COO classification

Assignment to non-GCB and GCB phenotype was performed based on immunohistochemical staining for CD10, BCL6, and MUM1. (Hans et al., 2004). When available (10/18 samples), total RNA was isolated from fresh-frozen biopsies using Allprep DNA/RNA mini kit (Qiagen). Gene expression profiling was performed on 100 ng RNA with a NanoString system and an extended custom-made probe set, covering 20 genes of the Lymph2Cx-assay for COO classification (Scott et al., 2014). Raw counts were uploaded to the Lymphoma/Leukemia Molecular Profiling Project website for COO categorization (https://llmpp.nih.gov/LYMPHCX/).

### BCR identification and expression vectors

Full-length V(D)J sequences of BCR heavy and light chain transcripts were identified and sequenced using unbiased ARTISAN PCR and direct Sanger sequencing (Koning et al., 2017). Sequence reads were analyzed with respect to variable gene usage and mutational status using Geneious 10.2.3 software and IMGT HighV-QUEST tools (Brochet et al., 2008). For ecotropic retroviral transduction of TKO cells with lymphoma-derived BCR, pMIZCC and pMIZYN vectors with human constant regions corresponding to isotype and light chain type expressed by individual lymphoma cases were synthesized (BaseClear BV).

### CRISPR/Cas9 gene editing

TMD8 CARD11^L251P/−^ cells were generated with CRISPR/Cas9 technology (Integrated DNA Technologies [IDT]). Ribonucleoproteins (RNP) were generated by annealing CRISPR RNA (crRNA):tracrRNA in a 1:1 ratio and complexes were formed with *Streptococcus pyogenes* (sp)Cas9 D10A nickase (IDT). A single stranded DNA oligo (ssODN) was designed to carry the L251P mutation, two protospacer adjacent motif–site silencing mutations, and a silent mutation creating a HindIII restriction site. RNPs and ssODN were cotransfected by electroporation into 2 × 10^6^ cells per reaction using the NEON transfection system (Thermo Fisher Scientific) with 100 μl tips in R-buffer using transfection settings: 1500 V 20 ms 2× pulses. Cells were single cell seeded in 384-well plates with limiting dilution (0.3 cells/well), and growing clones were screened. PCR products were amplified with Phusion Flash High-Fidelity PCR Master Mix (Thermo Fisher Scientific) with primers targeting *CARD11 *exon 5. Restriction of the PCR product was performed using HindIII (New England Biolabs). Clones with an incorporated restriction site were confirmed by Sanger sequencing. crRNA, ssODN, and primer sequences are shown in Table S3.

### Retroviral transduction

Ecotropic retroviral transduction particles were generated by transfecting vector constructs into Phoenix cells by liposomal transfection (FuGeneHD; Promega) accompanied with a pCL-help vector (RRID:Addgene_12371) (Naviaux et al., 1996). A separate supernatant was generated for every pMIZCC and pMIZYN construct. Supernatant containing viral particles was harvested 48 h after transfection. Double transduction of both heavy chain and light chain supernatant was performed on non-tissue culture treated plates (Greiner Bio) coated with 15 µg fibronectin (RN; Takara). After blocking with 2% human serum albumin, both supernatants were loaded on the plate and virus particles were forced to bind to RN by centrifugation for 30 min 4°C at 2,000 *g* before 1 × 10^5^ TKO cells were added. Transduction efficiency was measured for GFP expression by flow cytometry at 4–7 days after transduction.

### Ca^2+^ flux analysis

In addition to the transduced BCR, TKO cells express an ERT2-SLP65 dTomato-fusion protein. Upon treatment of 4-hydroxytamoxifen (4-OHT; Sigma-Aldrich; Merck), SLP65 is released from ERT2, activating the BCR signaling cascade leading to Ca^2+^ that is measured in the Ca^2+^ flux analysis, performed as described previously (Storch et al., 2007). In brief, 14–21 days after BCR transduction, 1 × 10^6^ cells were stained with 6 µg/ml Indo-1 (I1223; Thermo Fisher Scientific) diluted in calcium flux buffer composed of IMDM without Phenol red (21056; Gibco) supplemented with 1% FBS (P30-3302; PAN BioTech) for 45 min at 37°C. Until measurement, cells were kept in low volume at 4°C. Upon measurement, cells were resuspended in prewarmed (37°C) calcium flux buffer. Calcium-bound Indo-1 (∼404 nm) and calcium-free Indo-1 (∼480 nm) were measured over time. A baseline was measured and after 90 s calcium response was induced by 1 µM 4-OHT, measured for 450 s. Maximum response was measured for 90 s by addition of 10 µg/ml cognate antigen crosslinking antibodies (RRID:AB_2795610 and RRID:AB_2795657; SouthernBiotech). Measurements were performed on the LSRII (BD Biosciences) supported with Diva software and Aurora 5L (Cytek Europe) supported with Cytek software. Analysis was performed in FlowJo software (version 10.4.2). Signaling strength of the BCR was calculated as previously described (Quinten et al., 2023). In brief, the fraction of cells (Q^aut^) with a 405/485 signal intensity ratio (SIR) above the 95th percentile at baseline, i.e., prior to addition of 4-OHT, was determined with correction for totally unresponsive cells during BCR crosslinking measured in a 20-s bin. Subsequently, the median SIR observed during Q^aut^ was calculated and calibrated to the median SIR during the maximum response to BCR crosslinking (calSIR^aut^). Autonomous BCR signaling strength was quantified as the arithmetic product of Q^aut^ × calSIR^aut^. Signaling strength of every BCR was measured twice with an interval of 1–2 wk.

### Phospho-flow cytometry

Phosphorylation of BCR signaling proteins was performed on BCR-expressing TKO cells 14–21 days after transduction. On day −1, cells were seeded at a density of 1–2 × 10^5^ cells per well in a 96-well U-bottom plate. After a resting time of ∼18 h (overnight), cells were treated with 1 μM 4-OHT (Sigma-Aldrich) for 2 h. Cells were washed with PBS and stained with Zombie NIR (Biolegend) for 15 min at 37°C. Stimulation with crosslinking antibodies (RRID:AB_2795610 and RRID:AB_279565; SouthernBiotech) was done in half of the samples for 10 min and stopped by fixation for 20 min and permeabilization with the Foxp3/Transcription Factor Staining Buffer Set (Invitrogen; eBioscience; Thermo Fisher Scientific), followed by staining with phospho-flow antibodies AF647-labeled pCD79A Y182 (RRID:AB_2798979; Cell Signaling), AF647-labeled pBTK Y223 (RRID:AB_2721028; BD BioSciences), AF647-labeled pPLCγ2 Y759 (RRID:AB_647139; BD BioSciences), APC-labeled pSYK Y348 (Invitrogen; eBioscience), AF647-labeled pPI3K Y458/Y199 (RRID:AB_289529; Cell Signaling), or AF647-labeled pBLNK Y84 (RRID:AB_647111; BD BioSciences) for 20 min at room temperature while shaking. After washing, cells were measured with an LSR Fortessa flow cytometer (BD Biosciences). Data were analyzed with FlowJo software (version 10.4.2). After excluding dead cells using Zombie NIR, mean fluorescence intensity (MFI) was determined at 670 (± 15) in SLP65-ERT2- (dTomato) and BCR-expressing (GFP) double-positive cells. The MFI signal after BCR crosslinking was used to normalize the unstimulated samples.

### BCR genomic modification for growth support measurement

Testing of growth support capacity of BCR was performed by simultaneous replacement of the hypervariable region (HVR) sequences of light and heavy chains by homologous recombination-based knock-in into the U2932 cell line (Havranek et al., 2017). The knock-in also introduced a fluorescent marker of genomic modification (GFP for heavy chain modification and mTurqoise2 for light chain modification) preceding the HVR sequence and linked by an F2A sequence. To transfect the cells with pX330-U6-Chimeric_BB-CBh-hSpCas9 plasmids (Addgene) coding sgRNAs (Table S3) for U2932 BCR KO and repair template plasmids with desired HVR sequences (Fig. S2), cells were electroporated (Neon; Thermo Fisher Scientific). Cell growth was followed using non-fluorescent beads as a standard to estimate the absolute cell growth.

### Cell viability assay

BTK-inhibitor sensitivity was assessed with chromogenic proliferation assay (CellTiter 96 AQueous One Solution Cell Proliferation Assay; Promega). Cells were cultured for 4 days with a twofold titered concentration from 96 μM to 0.1875 μM, or equivalent volumes of the compound solvent DMSO. After 4 days, 20 μl of tetrazolium compound was added to each well, and after 4 h of incubation, OD^490^ values were measured using the SpectraMax M2e (Molecular Devices) and analyzed with Softmax Pro version 5.4.1. Samples were measured in duplicate per cell line (technical replicates). OD^490^ values were normalized to medium control.

### Genetic analyses

WES was performed on fragmented DNA with SureSelect Human All Exon V7 kit (Agilent) capture on the HiSeq2000 (Illumina) platform to an average coverage of 50×. For the variant calling analysis, FASTQ files were processed using the Sarek workflow v2.7 and aligned to the human reference genome GRCh38 using Burrows Wheeler Algorithm v0.7.17 (Garcia et al., 2020; Li and Durbin, 2009). Duplicated mapped reads were marked and local realignment of regions flanking indels and recalibration of base quality scores were performed to obtain more accurate bases according to the Genome Analysis ToolKit (GATK) best practices version v4.1.7.0 (McKenna et al., 2010). Single-nucleotide variants (SNV) and short insertions and deletions (INDELS) were called using Strelka2 v2.9.10 (Kim et al., 2018) Only high-confidence variants defined by Illumina's genotype quality score metric of at least 15 for SNV and 30 for INDELS were kept. The resulting variant call files were annotated by Ensembl-VEP (v103) with four filtering steps (McLaren et al., 2010). Variants were filtered for the NF-κB signaling pathway of the Kyoto Encyclopedia of Genes and Genomes (http://www.kegg.jp/entry/map04064) and the most frequently mutated genes in DLBCL (Chapuy et al., 2018; Kanehisa et al., 2023). Thereafter, variants were filtered by consequences, i.e., frameshift, in-frame deletion, missense, missense variant and splice region variant, splice region variant and synonymous variant, synonymous, in-frame insertion, stop gained, stop lost, frameshift variant and stop lost, missense variant and splice region variant, and coding sequence variant. Finally, variants were annotated for predicted effects by Combined Annotation Dependent Depletion phred, Sorting Intolerant From Tolerant (SIFT), and PolyPhen scores, and according to clinical impact. Benign variants annotated in Clinvar 202008 were discarded. Workflow quality control metrics were calculated and aggregated by MultiQC v1.8 (Ewels et al., 2016).

Recalibrated bam files from the variant calling workflow were used for detection of copy number variations (CNV) by the somatic copy number variation workflow following Broad’s recommended best practices using GATK4 CNV (McKenna et al., 2010) with a panel of 24 normal tissue samples. The modeled segment files were filtered by genomic coordinates of the genes/regions of interest (Chapuy et al., 2018).

Translocations of *MYC*, *BCL2*, and *BCL6* to the *IGH* locus or other genes of interest were identified by targeted locus capture-based sequencing (Allahyar et al., 2021).

### Assignment to molecular DLBCL clusters

Assignment of individual cases to LymphGen clusters was performed by uploading the available WES sequence, CNV, and translocation information to the LymphGen data portal (https://llmpp.nih.gov/lymphgen/index.php) (Wright et al., 2020). Assignment to consensus clusters of each individual case was calculated by conditional probabilities based on the observed sequence characteristics (wild-type versus aberrant) of cluster-defining loci in relation to the described frequencies of these characteristics used for the definition of the clusters (Chapuy et al., 2018).

### Online supplemental material

Fig. S1 shows gating and quality control of flow cytometry with respect to calculation of BCR signaling strength. Fig. S2 shows the sequence alignment of closely related and modified VDJ sequences used for functional testing. Table S1 includes the BCR characteristics of ABC-DLBCL cell lines and 18 DLBCL cases. Table S2 lists the variants in genes of the NF-κB pathway and genes relevant for genetic clusters of DLBCL. Table S3 specifies sequences of primers, sgRNAs, and an ssODN template for CRISPR/Cas9 gene editing.

## Supplementary Material

Table S1 shows the BCR characteristics of ABC-DLBCL cell lines and 18 DLBCL cases.

Table S2 shows the variants in genes of the NF-κB pathway and genes relevant for genetic clusters of DLBCL.

Table S3 specifies sequences of primers, sgRNAs, and an ssODN template for CRISPR/Cas9 gene editing.

## Data Availability

The data supporting the findings of this study are available within the article and the main figures or its supplementary materials. Raw flow cytometry data demonstrated in the figures and used to generate plots are available upon request from the corresponding author (H. Veelken). WES sequencing data are publicly available in Dryad: https://doi.org/10.5061/dryad.612jm647m.
