# Supplementary material for: Antigen-independent, autonomous B cell receptor signaling drives activated B cell DLBCL
Source: J Exp Med. 2024 Mar 21;221(5):e20230941. doi: 10.1084/jem.20230941 (PMC10959178; doi:10.1084/jem.20230941)
Supplement: Table S1 — shows the BCR characteristics of ABC-DLBCL cell lines and 18 DLBCL cases. [file jem_20230941_tables1.docx]

**Supplementary Table 1:** **BCR characteristics of ABC-DLBCL cell lines and 18 DLBCL cases**

| **Cell line / Case** | **IGHV** | **IGHV identity to GL** | **VJ Junction** | **HCDR3 length** | **IGHJ** | **Isotype** | **I/VRQ motif FR2** | **YYC motif FR3** | **IGLV** |
| --- | --- | --- | --- | --- | --- | --- | --- | --- | --- |
| **TMD8** | IGHV3-48*02 | 87.50% | CVRDRMGGNPW | 9 | IGHJ5*02 | IgM | VRQ | YYC | IGKV2-30 |
| **OCI-Ly3** | IGHV4-34*01 | 83.51% | CARGRTGDAEGDVAGLGYYYDFW | 21 | IGHJ4*02 | IgG | IRQ | YYC | IGLV3-21 |
| **Karpas 422** | IGHV4-39*07 | 84.54% | CAKNSSVPIVRGLNKRYSFFDLW | 21 | IGHJ6*04 | IgG | IRQ | YYC | IGK2D-28 |
|  |  |  |  |  |  |  |  |  |  |
| **3872** | IGHV2-5*04 | 80.07% | CGHQTYDRAWYPFISW | 14 | IGHJ4*02 | IgM | IRH | YFC | IGKV1-12 |
| **5244** | IGHV2-70*04 | 87.63% | CARTSNIGTYTVVADHFDSW | 18 | IGHJ1*01 | IgM | IRQ | YFC | IGLV1-51 |
| **2997** | IGHV3-23*01 | 96.18% | CAKKTESGSLTPFDYW | 14 | IGHJ4*02 | IgM | VRQ | YYS | IGKV4-1 |
| **3315** | IGHV3-23*01 | 92.01% | CARHNWQMNGIYYNGLDVW | 17 | IGHJ6*02 | IgM | VRQ | YYC | IGKV2-28 |
| **4609** | IGHV3-33*03 | 70.83% | CAAQTSGLDFW | 9 | IGHJ4*02 | IgM | VRQ | YYC | IGKV3-15 |
| **3844** | IGHV3-48*02 | 82.99% | CTLGGALDVW | 8 | IGHJ3*01 | IgM | VRQ | YYC | IGKV2-24 |
| **3166** | IGHV3-48*02 | 85.07% | CVRDLLLTHW | 8 | IGHJ4*02 | IgM | VRQ | YYC | IGKV2-30 |
| **5252** | IGHV3-7*01 | 84.03% | CVSFDQGLMSADPW | 12 | IGHJ5*02 | IgM | VRQ | YYC | IGLV1-44 |
| **4760** | IGHV3-74*01 | 74.74% | CARHRNTYTFDMW | 11 | IGHJ3*02 | IgM | VRQ | YHC | IGKV1-33 |
| **208** | IGHV3-74*01 | 87.50% | CARGKNTNFIDFW | 11 | IGHJ4*02 | IgM | VRQ | YYC | IGKV3-11 |
| **3752** | IGHV4-34*01 | 88.77% | CARGTPLTSSSESYFHFDVW | 18 | IGHJ6*03 | IgM | IRQ | YYC | IGLV3-19 |
| **3267** | IGHV4-34*02 | 85.96% | CARGPSSSLSLLKTGSGVDVW | 19 | IGHJ6*02 | IgM | IRQ | FFC | IGLV2-11 |
| **3217** | IGHV4-34*02 | 88.77% | CARGGHDYGDYKPQEFDCW | 17 | IGHJ4*02 | IgM | VRQ | YYC | IGLV7-43 |
| **3882** | IGHV4-39*01 | 93.47% | CARHGPSGNNYFFDYW | 14 | IGHJ4*02 | IgG1 | IRQ | YYC | IGKV3D-20 |
| **4391** | IGHV3-74*01 | 88.54% | CVRDLAGRWGTW | 10 | IGHJ3*02 | IgG2 | VRQ | YYC | IGLV1-51 |
| **3567** | IGHV1-58*01 | 92.01% | CAADPGRAYDIHRDSYHYYYGLDVW | 23 | IGHJ6*02 | IgG2 | VRQ | FYC | IGLV1-47 |
| **3850** | IGHV1-69*01 | 67.71% | CARLQGGTIEVSAFDSW | 15 | IGHJ4*02 | IgG4 | VRQ | YFC | IGKV4-1 |
| **4328** | IGHV3-23*01 | 81.94% | CVVDRRSDSGDWSSHW | 14 | IGHJ5*02 | IgM | VRQ | YYC | IGKV4-1 |
