# Supplementary material for: Antigen-independent, autonomous B cell receptor signaling drives activated B cell DLBCL
Source: J Exp Med. 2024 Mar 21;221(5):e20230941. doi: 10.1084/jem.20230941 (PMC10959178; doi:10.1084/jem.20230941)
Supplement: Table S3 — specifies sequences of primers, sgRNAs, and an ssODN template for CRISPR/Cas9 gene editing. [file jem_20230941_tables3.docx]

**Supplementary Table S3: Oligonucleotide sequences**

| **Name** | **Target** | **Sequence** |
| --- | --- | --- |
| Exon 5 forward primer * | CARD11 | 5’ - CAGAGGAGGAGACAAAGGTGCC |
| Exon 5 reverse primer | CARD11 | 5’ - TTCTCTGCCCGTGGAAGAAGG |
| crRNA L251P_1 | CARD11 | 5’ - CCTCCTCCATCTTATTCAAC |
| crRNA L251P_2 | CARD11 | 5’ - TGAAGAATGACATTGAAAAT |
| ssODN | CARD11 | 5’ - TGGGGTATTTCAGATCGATCAGCTAAAGCACAGGTTGAATAAGATGGAGGAGGAATGTAAGCTTGAG AGAAATCAGTCTCCAAAACTGAAGAATGACATTGAAAATCGTCCCAAGAAGGAGCAGGTTCTGGAACTGGA |
| sgRNA_1 | U2932 BCR | 5’ - GCAAGAAAATGAAGCACCTGTGG |
| sgRNA_2 | U2932 BCR | 5’ - AAAGCAGGAGAGAGGTCGTGAGG |

*used for sequencing
